# Association between the Phytochemical Index and Overweight/Obesity: A Meta-Analysis

**DOI:** 10.3390/nu14071429

**Published:** 2022-03-29

**Authors:** Chaojie Wei, Liping Liu, Renli Liu, Wenwen Dai, Weiwei Cui, Dong Li

**Affiliations:** 1 Department of Immunology, College of Basic Medical Sciences, Jilin University, 126 Xinmin Avenue, Changchun 130021, China; weicj21@mails.jlu.edu.cn (C.W.); liulp21@mails.jlu.edu.cn (L.L.); liurl20@mails.jlu.edu.cn (R.L.); 2 Department of Nutrition and Food Hygiene, School of Public Health, Jilin University, 1163 Xinmin Avenue, Changchun 130021, China; daiww20@mails.jlu.edu.cn

**Keywords:** phytochemical index, overweight, obesity, meta-analysis, dose–response relationship

## Abstract

Some studies suggest that a higher phytochemical index (PI) is associated with a lower risk of overweight/obesity. This meta-analysis is performed to summarize published studies on the relationship of PI and the risk of overweight/obesity. We searched on PubMed, Cochrane Library and Web of Science from the inception dates to February 2022. The random-effect model was used based on heterogeneity. Meta-regression was used to explore potential sources of between-study heterogeneity. Publication bias was evaluated using Begg’s and Egger’s tests. The dose–response relationship was assessed using a restricted cubic spline model. Nine studies were included in the meta-analysis, with a total of 100,753 participants. The meta-analysis showed that the phytochemical index was associated with a decreased risk of overweight/obesity. The pooled OR (95% CI) was 0.81 (0.74–0.90). The findings from dose–response analysis showed a nonlinear association between the phytochemical index and the risk of overweight/obesity. The results of the meta-regression showed that gender and area were significant covariates influencing the heterogeneity between studies. There was no publication bias in the meta-analysis of this study. In conclusion, although this meta-analysis indicates that a high phytochemical index is associated with a reduced risk of overweight/obesity, all the studies included in this meta-analysis were cross-sectional studies with high heterogeneity. As such, more data from randomized controlled trials are required to confirm the efficacy of PI in evaluating the risk of overweight/obesity.

## 1. Introduction

Overweight and obesity are characterized by abnormal or excessive fat accumulation that can damage health [1]. Recent statistics indicate that overweight/obesity continues to increase worldwide, with more than 2 billion overweight people, representing approximately 30% of the world’s population [2]. The Global Burden of Disease Group reported in 2017 that the prevalence of obesity has doubled in more than 70 countries since 1980 and continues to increase in most other countries [3]. Obesity is mainly caused by lifestyle, genetic and environmental factors [4]. As a modifiable behavior, eating behavior plays an important role in the etiology of obesity [5].

Several epidemiological and cross-sectional studies have shown that vegetarian diets can reduce body weight and body mass index (BMI) [6,7,8,9]. In addition, there is evidence that plant-based diets are associated with the prevention and treatment of chronic diseases [10,11]. The phytochemical index/dietary phytochemical index (PI/DPI) is the percentage of total dietary calories derived from foods rich in phytochemicals, such as fruits, vegetables (except potatoes with few phytochemicals), legumes, nuts, seeds and whole grains. PI is a simple way of assessing the intake of phytochemicals to effectively assess the health effects of foods rich in phytochemicals [12]. Phytochemicals, the physiologically active substances commonly found in plant-based foods such as fruits, vegetables, grains and legumes [13], can help improve chronic diseases such as diabetes, cardiovascular disease and some cancers [14]. Phytochemicals, mainly including polyphenols, alkaloids, terpenoids, flavonoids, saponins, steroids and so on, can prevent obesity by regulating carbohydrate and lipid metabolism [15].

Some studies have suggested that a higher phytochemical index (PI) is associated with a lower risk of overweight/obesity [5,16,17,18,19,20]. However, no study has comprehensively analyzed the relationship between PI and overweight/obesity. Therefore, this meta-analysis was performed to summarize published studies on the relationship between PI and the risk of overweight/obesity.

## 2. Materials and Methods

### 2.1. Sources and Methods of Data Retrieval

We searched PubMed, the Cochrane Library and Web of Science from the inception dates to February 2022. The following keywords were used to identify published literature which examined the relationship between PI and the incidence of overweight or obesity: phytochemicals, dietary phytochemicals, plant bioactive compounds, plant biologically active compounds, plant-derived chemicals, phytonutrients, non-nutrient bioactive substances, bioactive food components, index, overweight, and obesity.

### 2.2. Inclusion Criteria and Exclusion Criteria

The included articles needed to meet the following five inclusion conditions: (1) The exposure variable was PI/DPI; (2) the outcome variables were overweight/obesity or centripetal obesity; (3) the literature needed to provide an odds ratio/relative risk/hazard ratio (OR/RR/HR) and a 95% confidence interval (CI) between PI and overweight/obesity; (4) the literature used for dose–response analysis must provide the dose, number of cases, number of person-years in each group, or the data can be obtained by the conversion of missing values; and (5) the language of the original article was English. The following two exclusion criteria were applied: (1) the study subjects were not humans and (2) review.

### 2.3. Data Extraction and Quality Assessment

Data were independently extracted and cross-checked by two researchers according to uniform standards, and experts could be consulted in case of disagreement. The main contents of data extraction included: first author, publication year, area, dose, cases per year and OR/RR/HR in each exposure–dose range.

The quality of the included literature was assessed using the 11-item checklist recommended by the Healthcare Research and Quality Authority (AHRQ). Article quality was assessed as follows: low quality = 0–3; moderate quality = 4–7; and high quality = 8–11 [21].

### 2.4. Statistical Analysis

Stata12.0 software was used for statistical analysis, the Q test was used to test the heterogeneity of the included studies, I2 was used to evaluate the heterogeneity, and the test level was α = 0.05. If I^2^ < 50% [22], there was no statistical heterogeneity between studies. A fixed-effect model was used to calculate the combined effect OR and 95% CI. If I^2^ ≥ 50% [23], the random-effects model was used. Meta-regression was used to explore the potential sources of inter-study heterogeneity. Sensitivity analysis was used to test the stability of the results. The Begg and Egger methods [24,25] were used to evaluate publication bias. The dose–response relationship was evaluated using the restricted cubic spline model [26].

## 3. Results

### 3.1. Study Characteristics

**Figure 1:**
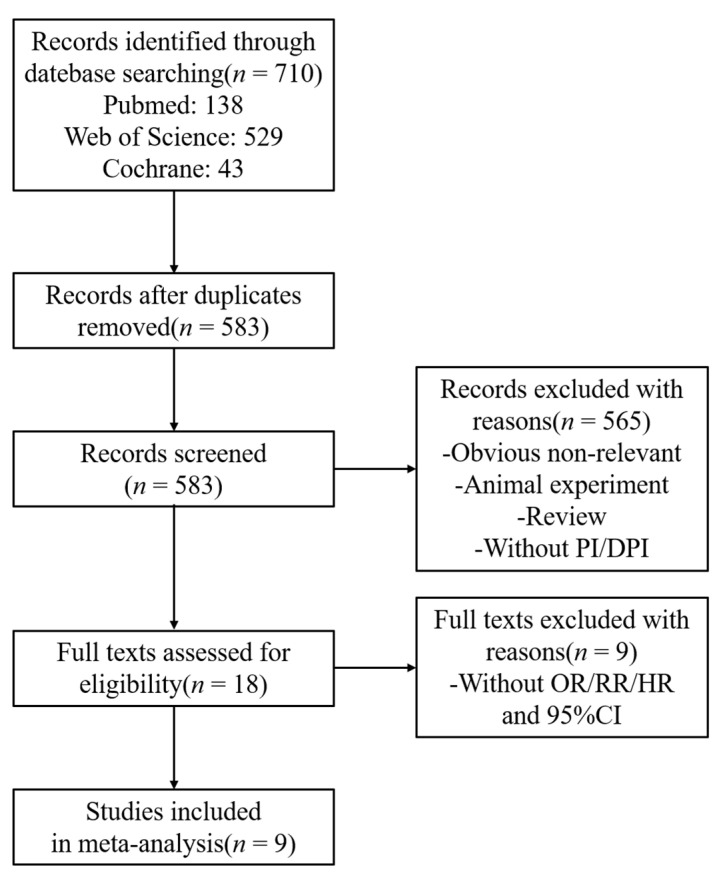
Flow chart of study selection. Abbreviations: PI: phytochemical index; DPI: dietary phytochemical index; OR: odds ratio; RR: relative risk; HR: hazard ratio; CI: confidence interval.

The literature-screening process is shown in Figure 1. We initially screened out 710 studies and, after the removal of duplicates, according to the inclusion and exclusion criteria formulated in this study, nine studies [5,16,17,18,19,20,27,28,29] including 22 groups of data were finally included, with a total of 100,753 participants. The included studies were scored by the AHRQ scale and all were of moderate quality. The characteristics and quality scores of the selected studies are presented in Table 1.

### 3.2. Meta-Analysis

**Figure 2:**
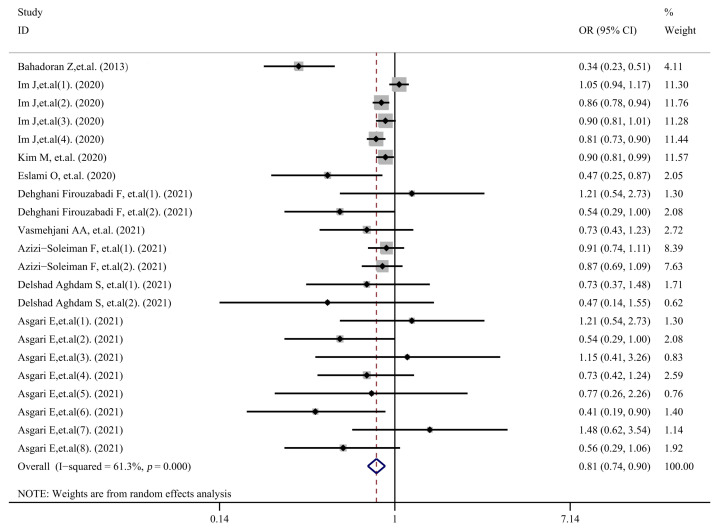
Meta-analysis of PI (comparing the highest with the lowest PI categories) and risk of overweight/obesity [5,16,17,18,19,20,27,28,29].

Figure 2 shows the multivariable-adjusted ORs for the highest versus lowest categories of PI. The meta-analysis shows that subjects in the highest category of PI had a significantly decreased risk for overweight/obesity, compared with those in the lowest category. The pooled OR was 0.81 (95% CI: 0.74–0.90) and high heterogeneity was observed (I^2^ = 61.3%; *p* < 0.001).

### 3.3. Subgroup Analysis

**Figure 3:**
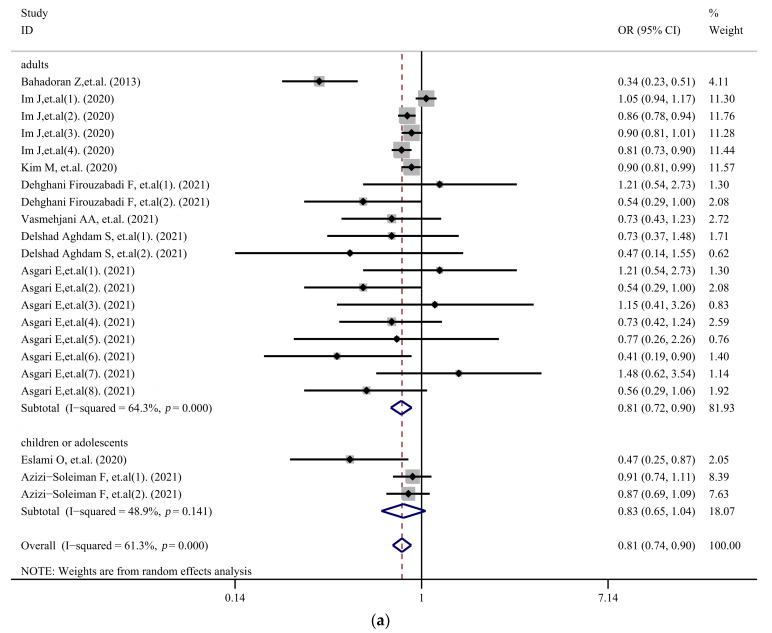

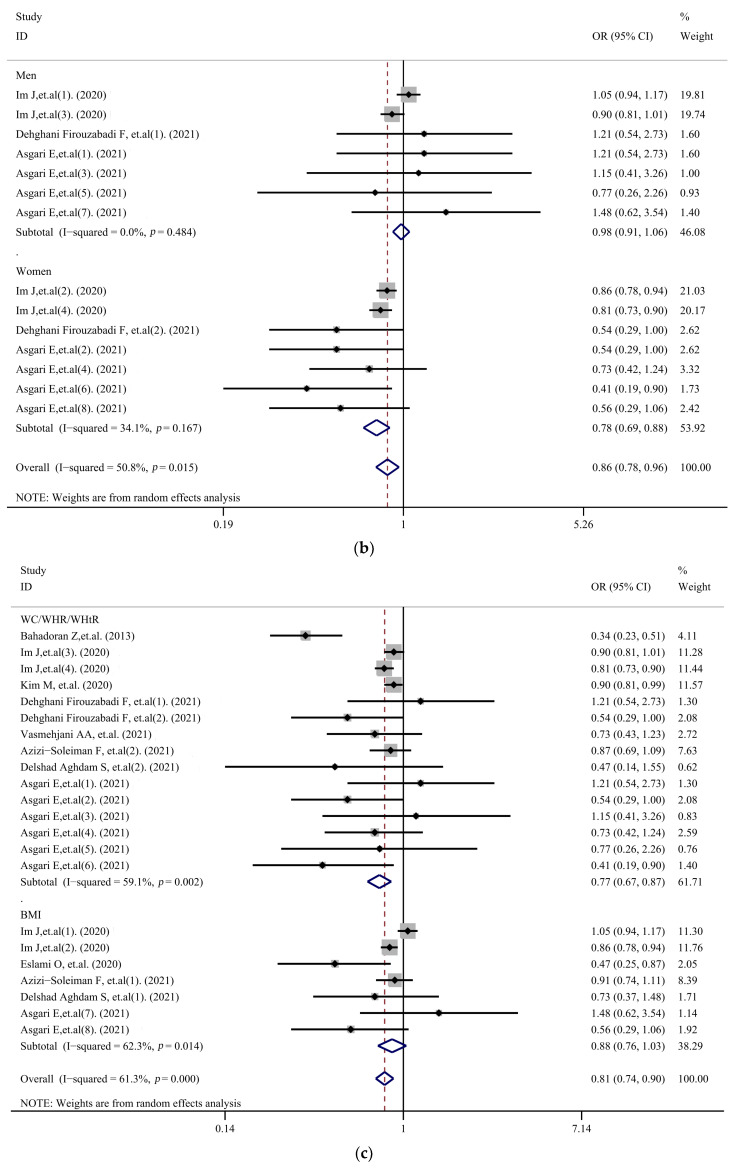

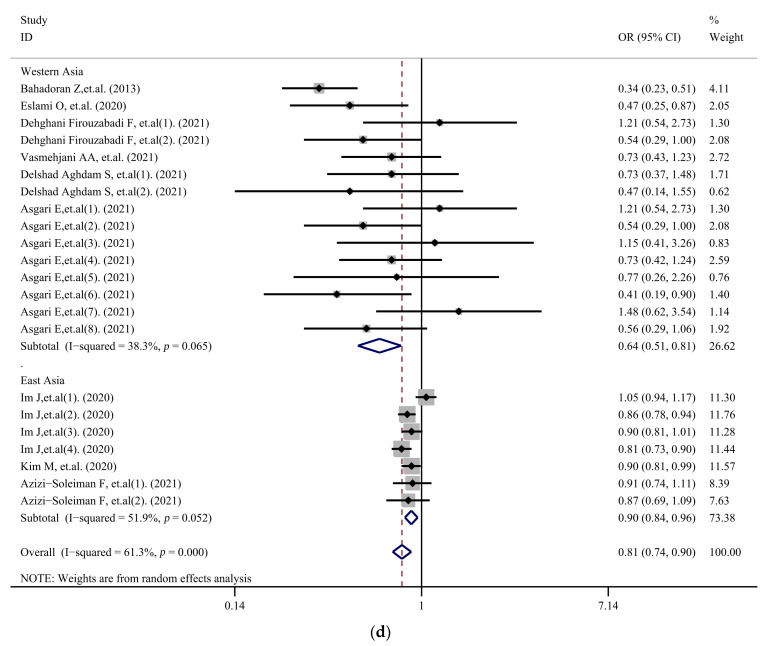
Subgroup analysis stratified by (**a**) age; (**b**) gender; (**c**) diagnostic criteria of overweight/obesity; and (**d**) area [5,16,17,18,19,20,27,28,29].

Figure 3 shows the results of the subgroup analysis. The subgroup analysis of age showed that the highest PI was a protective factor in adults (OR, 0.81; 95% CI, 0.72–0.90; I^2^ = 64.3%, *p* < 0.001); however, there was no statistically significant relationship between the highest PI and overweight/obesity in children or adolescents (OR, 0.83; 95% CI, 0.65–1.04; I^2^ = 48.9%, *p* = 0.141). In addition, a total of three studies [17,19,20] including 14 sets of data were used to study the relationship between PI and obesity incidence for different genders. Therefore, subgroup analysis by gender was performed on these data, which showed that PI was associated with the prevalence of overweight/obesity in women (OR, 0.78; 95%CI, 0.69–0.88; I^2^ = 34.1%, *p* = 0.167), but not in men (OR, 0.98; 95%CI, 0.91–1.06; I^2^ = 0, *p* = 0.484). The subgroup analysis of diagnostic criteria of overweight/obesity showed that PI was related to the prevalence of overweight/obesity when waist circumference/waist-to-hip ratio/waist-to-height ratio (WC/WHR/WHtR) was used to determine obesity (OR, 0.77; 95%CI, 0.67–0.87; I^2^ = 59.1%, *p* = 0.002), but this was not found when BMI was used (OR, 0.88; 95%CI, 0.76–1.03; I^2^ = 62.3%, *p* = 0.014). A subgroup analysis of area showed that the highest PI was associated with overweight/obesity in both West Asia (OR, 0.64; 95%CI, 0.51–0.81; I^2^ = 38.3%, *p* = 0.065) and East Asia (OR, 0.90; 95%CI, 0.84–0.96; I^2^ = 51.9%, *p* = 0.052).

### 3.4. Sensitivity Analysis

The included studies were removed one by one, and the remaining studies were meta-analyzed. The results showed that the combined effect value changed greatly and heterogeneity decreased (I^2^ = 38.4%) after the first article [16] was removed, indicating that this article might be the source of heterogeneity (Table 2).

### 3.5. Meta-Regression

In order to explore the source of heterogeneity, we conducted a meta-regression analysis with age, gender, diagnostic criteria of obesity and area (West or East Asia) as covariates (Table 3). The results showed that gender and area were significant covariates influencing the heterogeneity between studies. Other covariables were not shown to have a significant effect on inter-study heterogeneity.

### 3.6. Publication Bias

**Figure 4:**
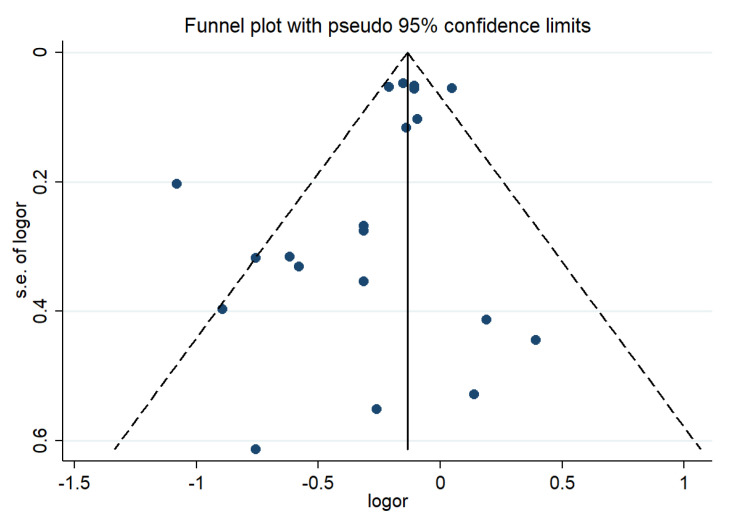
Funnel plot of the associations between PI and overweight/obesity. Abbreviations: s.e.: standard error; logor: the logarithm of OR.

A funnel plot (Figure 4), Begg’s test and Egger’s test were used to detect publication bias, and no publication bias was found (*p* for Begg’s test = 0.955; *p* for Egger’s test = 0.059).

### 3.7. Dose–Response Analysis

**Figure 5:**
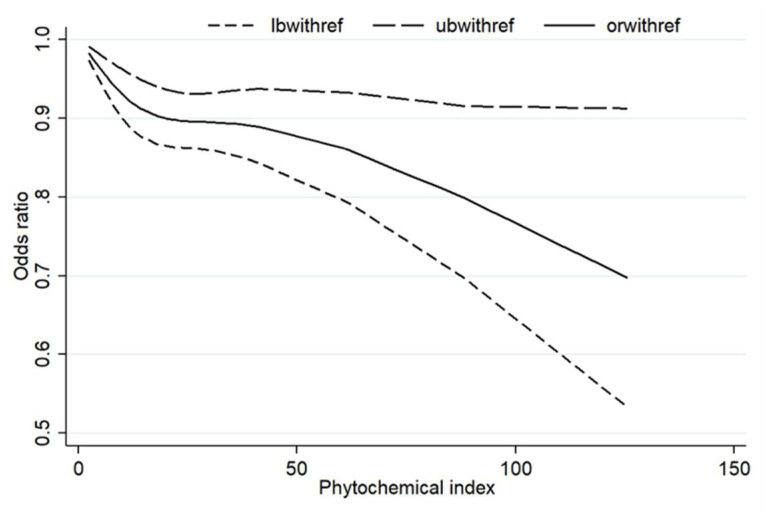
The dose–response analysis between PI and the risk of overweight/obesity. Abbreviations: lbwithref: lower bound of 95% confidence interval with reference; ubwithref: upper bound of 95% confidence interval with reference; orwithref: odds ratio with reference.

We included four articles including 13 sets of data to study the dose–response relationship (Figure 5). The results showed that there was a nonlinear dose–response relationship between PI and the risk of overweight/obesity (*p* = 0.0447).

## 4. Discussion

A growing number of studies have investigated the effects of the phytochemical index on chronic diseases such as metabolic syndrome [18,19], diabetes [30], cardiovascular disease [29] and breast-related diseases [31,32,33]. Phytochemicals also have anti-obesity properties. However, relevant studies reported controversial results for the relationship between the phytochemical index and obesity risk. We conducted a meta-analysis to quantify previous studies. This meta-analysis showed a possible inverse association between a higher PI and the risk of overweight/obesity, which was consistent with a longitudinal study on adults, reporting that increasing energy intake from phytochemical-rich foods can prevent weight gain and aid weight loss in adults [34].

From the results of the subgroup analysis, there are several factors worth considering. First, the results varied according to the age of the participants in the original study. A subgroup analysis of age showed that a higher PI was a protective factor in adults. However, the results were not statistically significant in children or adolescents, which may be due to the lack of corresponding literature. To date, only a few studies have assessed the link between PI and obesity, and most of the research was conducted on adults.

Subgroup analysis by gender found that women with a higher PI had a lower risk of obesity, but no association between PI and obesity prevalence was observed in men. In fact, a study of adults in Korea found that a higher intake of total flavones was associated with a lower risk of obesity; however, this association was not found in men [35]. In addition, an epidemiological study found an association between serum carotenoid levels and abdominal obesity in women, but no corresponding significant association was found in men [36]. This may be due to the interaction between phytochemicals and sex hormones. Certain types of phytochemicals have structures similar to estrogen and can mimic or influence the effects of estrogen in the body. Therefore, the intake of these phytochemicals may improve diseases caused by estrogen deficiency [37,38,39,40]. That is, the intake of phytochemical-rich foods may reduce the obesity rate by helping the female body improve hormone levels.

A subgroup analysis using diagnostic criteria of overweight/obesity found that when obesity was defined by WC/WHR/WHtR, the incidence of overweight/obesity was lower in people with a high PI. A cross-sectional survey of 54 adults showed a negative correlation between DPI and WC. Phytochemicals may inhibit preadipocyte proliferation through partial polyphenols, reduce adipogenesis and promote lipid decomposition to maintain WC normality [15,41]. A subgroup analysis by area showed a negative association between high PI and obesity incidence, but the association was more obvious in the West Asian group, possibly because most of the studies were conducted in Iran. In addition, there are no studies on PI and obesity outside of Asia, which may be due to dietary differences among regions. Given these differences, further studies on the role of phytochemicals in obesity are needed to clarify the causal relationship.

In addition, the dose–response relationship showed a decreased risk of overweight/obesity with increased PI. Diets with more phytochemicals are generally lower in calories, so they are more likely to reduce obesity risk. Some studies have shown that a diet rich in phytochemicals can improve obesity by reducing oxidative stress, inducing the production of pro-inflammatory cytokines, promoting thermogenesis, inhibiting adipocyte differentiation and reducing adipogenesis [42,43].

Inter-study heterogeneity is a key issue in meta-analysis, which directly affects the interpretation of meta-analysis results. Therefore, it is an important aspect of this study to explore the potential sources of heterogeneity between studies. The results of our meta-regression showed that gender and area were the significant covariables affecting inter-study heterogeneity. The sensitivity analysis results showed that after removing Bahadoran Z’s study [16], the heterogeneity was reduced to 38.2%, suggesting that this study may be the source of the heterogeneity. This is thought to be because it is based on survey data from 2006 to 2008, which is early compared with other studies. In addition, there were more elderly people with high PI scores in this study. The above may be the cause of the heterogeneity. Begg’s and Egger’s tests showed that there was no publication bias in the meta-analysis of this study.

## 5. Conclusions

Our meta-analysis showed that PI was inversely associated with the risk of overweight/obesity. With the increase in PI, the prevalence of overweight/obesity decreased gradually. However, these findings were limited because the studies included in this meta-analysis were all cross-sectional studies. Therefore, no definite conclusions can be drawn at present. Due to the high heterogeneity of cross-sectional studies, this evidence needs further validation.

## Figures and Tables

**Table 1:** Characteristics of the studies included.

Study	Country	Age	Subjects	Outcome Variable	Diagnostic Criteria	Score
Bahadoran Z, 2013 [16]	Iran	19–70	2567	Abdominal obesity	WC ≥ 95 cm	7
Im J, 2020 [17]	Korea	≥19	57,940	Obesity, abdominal obesity	BMI ≥ 25 kg/m^2^, WC ≥ 90 and ≥85 cm for men and women	7
Kim M, 2020 [18]	Korea	≥19	31,319	Abdominal obesity	WC ≥ 90 cm in men and ≥80 cm in women	6
Eslami O, 2020 [5]	Iran	7–10	356	Overweight and obesity	overweight: BMI percentile ≥ 85 and <95, obese: ≥95	6
Dehghani Firouzabadi F, 2021 [19]	Iran	18–65	844	Central obesity	WC ≥ 102 cm for men and 88 cm for women	7
Vasmehjani AA, 2021 [27]	Iran	20–70	2326	Abdominal obesity	WC ≥ 102 cm for men and >88 cm for women	6
Azizi-Soleiman F, 2021 [28]	China	6–18	4296	Obesity or overweight, abdominal obesity	BMI > 85th percentile, WHtR ≥ 0.5	7
Delshad Aghdam S, 2021 [29]	Iran	18–35	261	Overweight or obesity, abdominal obesity	BMI > 24.9 kg/m^2^, WC ≥ 80 cm in women and ≥94 cm in men	6
Asgari E, 2021 [20]	Iran	18–59	844	Central obesity, general obesity	BMI ≥ 30 kg/m^2^, central obesity: WHtR ≥ 0.5; WHR ≥ 0.8 for women and ≥1 for men; WC ≥ 102 cm for men and ≥88 cm for women	7

Abbreviations: BMI: body mass index; WC: waist circumference; WHR: waist-to-hip ratio; WHtR: waist-to-height ratio. Score was rated using an 11-item checklist that was recommended by the Agency for Healthcare Research and Quality.

**Table 2:** Sensitivity analysis by removing one by one the included studies.

Study	OR (95%CI)	I^2^	*p*
Bahadoran Z, et al. (2013) [16]	0.87 (0.80–0.93)	38.4%	0.039
Im J, et al. (1). (2020) [17]	0.79 (0.72–0.87)	51.9%	0.003
Im J, et al. (2). (2020) [17]	0.80 (0.71–0.89)	63.1%	<0.001
Im J, et al. (3). (2020) [17]	0.80 (0.71–0.89)	63.0%	<0.001
Im J, et al. (4). (2020) [17]	0.81 (0.72–0.90)	61.4%	<0.001
Kim M, et al. (2020) [18]	0.79 (0.71–0.89)	62.9%	<0.001
Eslami O, et al. (2020) [5]	0.83 (0.75–0.91)	60.4%	<0.001
Dehghani Firouzabadi F, et al. (1). (2021) [19]	0.81 (0.73–0.89)	62.8%	<0.001
Dehghani Firouzabadi F, et al. (2). (2021) [19]	0.82 (0.75–0.91)	61.5%	<0.001
Vasmehjani AA, et al. (2021) [27]	0.82 (0.74–0.90)	62.9%	<0.001
Azizi-Soleiman F, et al. (1). (2021) [28]	0.80 (0.72–0.89)	63.1%	<0.001
Azizi-Soleiman F, et al. (2). (2021) [28]	0.81 (0.73–0.89)	63.2%	<0.001
Delshad Aghdam S, et al. (1). (2021) [29]	0.81 (0.74–0.90)	63.0%	<0.001
Delshad Aghdam S, et al. (2). (2021) [29]	0.82 (0.74–0.90)	62.5%	<0.001
Asgari E, et al. (1). (2021) [20]	0.81 (0.73–0.89)	62.8%	<0.001
Asgari E, et al. (2). (2021) [20]	0.82 (0.75–0.91)	61.5%	<0.001
Asgari E, et al. (3). (2021) [20]	0.81 (0.73–0.89)	63.0%	<0.001
Asgari E, et al. (4). (2021) [20]	0.82 (0.74–0.90)	62.9%	<0.001
Asgari E, et al. (5). (2021) [20]	0.81 (0.74–0.90)	63.1%	<0.001
Asgari E, et al. (6). (2021) [20]	0.82 (0.75–0.91)	60.5%	<0.001
Asgari E, et al. (7). (2021) [20]	0.81 (0.73–0.89)	62.2%	<0.001
Asgari E, et al. (8). (2021) [20]	0.82 (0.74–0.90)	61.9%	<0.001
Combined	0.81(0.74–0.90)	61.3%	<0.001

**Table 3:** Meta-regressions by age, gender, diagnostic criteria of obesity and area.

Covariate	*p*
Age	0.895
Gender	0.023
Diagnostic criteria of obesity	0.360
Area	0.002
